# Effects of gatekeeper trainings from the Austrian national suicide prevention program

**DOI:** 10.3389/fpsyt.2023.1118319

**Published:** 2023-07-20

**Authors:** Martin Plöderl, Clemens Fartacek, Thomas Kapitany, Ulrike Schrittwieser, Thomas Niederkrotenthaler

**Affiliations:** ^1^Center for Inpatient Psychotherapy and Crisis Intervention, Department of Psychiatry, Psychotherapy and Psychosomatics, Christian-Doppler University Hospital, Paracelsus Medical University, Salzburg, Austria; ^2^Department of Clinical Psychology, Department of Psychiatry, Psychotherapy and Psychosomatics, Christian-Doppler University Hospital, Paracelsus Medical University, Salzburg, Austria; ^3^Crisis Intervention Center Vienna, Vienna, Austria; ^4^Institute for Suicide Prevention, Graz, Austria; ^5^Suicide Research Unit, Department of Social and Preventive Medicine, Center for Public Health, Medical University of Vienna and Wiener Werkstaette for Suicide Research, Vienna, Austria

**Keywords:** suicide, prevention, gatekeeper, training, skills, SUPRA

## Abstract

**Background:**

The development and implementation of gatekeeper trainings were first goals in the national suicide prevention strategy “Suicide Prevention Austria” (SUPRA). The current study aims to assess the short- and longer-term effects of the SUPRA gatekeeper trainings in comparison with established gatekeeper trainings.

**Methods:**

We evaluated 28 gatekeeper trainings including 427 participants by assessing improvement of knowledge (facts about suicide and suicide prevention), gatekeeper self-efficacy and attitudes, and gatekeeper behavior (e.g., asking depressed people about suicide). Assessments were immediately before and after the gatekeeper trainings, with an additional follow-up 6 months later. Effects were compared with benchmark effects of established gatekeeper trainings.

**Results:**

There were substantial improvements in knowledge, self-efficacy and attitudes immediately after the training, comparable or larger than known from evaluations of established gatekeeper trainings. Most of these changes were upheld in the follow-up assessment, with effects comparable to other gatekeeper trainings. There was only a small increase of self-reported gatekeeper behavior, in line with results from other gatekeeper trainings.

**Conclusion:**

The SUPRA gatekeeper training had some beneficial effects in the short- and longterm, with effect sizes comparable to established gatekeeper trainings.

## 1. Introduction

With more than 700,000 annual deaths worldwide, suicidal behavior is a public health problem, making suicide prevention a global imperative (1). There are a number of suicide-specific interventions that demonstrate a reduction in suicide risk (2). However, access to effective intervention seems to be limited, unfortunately, because the majority of suicide decedents did not have contact with a mental health provider in the year and even fewer within in the month before death (3, 4). Therefore, one major goal of suicide prevention is gatekeeping, that is, getting individuals at elevated risk for suicide into contact with the appropriate professional health services (5). Consequently, these gatekeeper strategies are often part of national/organizational suicide prevention strategies (6). Gatekeepers are persons who, due to their professional or social role, encounter people potentially at risk for suicide and are able to identify them by recognizing suicide risk warning signs, offer appropriate help, and referring to professional help. To improve gatekeeper-behavior, gatekeeper trainings typically focus on knowledge (e.g., warning signs for suicide), attitudes (e.g., beliefs suicide is considered preventable), and skills (e.g., assessment of suicidality) (7).

Studies about the effects of gatekeeper trainings predominantly focus on knowledge, self-efficacy, attitudes, and self-reported gatekeeper behavior, because an objective and direct assessment of gatekeeper behavior and its impact on suicidal behavior in the target population is challenging. Studies found that gatekeeper-relevant knowledge improved in the short- and long-term (8, 9). However, knowledge may have little impact on the intervention behavior of gatekeepers (10). According to the theory of planned behavior, the likelihood of actual behavior is predicted by attitudes, perceived behavioral control, and subjective norms (11). Therefore, typical studies also investigated the impact of gatekeeper trainings on attitudes and perceived behavioral control, that is, the gatekeeper’s perception of his or her ability to perform the gatekeeper behavior (also referred to as gatekeeper self-efficacy). While substantial positive effects for gatekeeper self-efficacy were found in the short- and long-term, there was mixed evidence for change of attitudes (8, 9). For self-reported gatekeeper behavior the effects were generally small (8, 9).

In 2012, Austria founded its national suicide prevention strategy “Suizidprävention Austria” (SUPRA), coordinated by the ministry of health, and developed by a group of experts from all Austrian counties (12). The development and nationwide implementation of gatekeeper trainings were important early goals. The SUPRA gatekeeper training program was developed based on the experience of a long-standing Austrian tradition of gatekeeper trainings and on existing international concepts adapted to national and regional circumstances (e.g., knowledge of regional care services, language, cultural differences, theoretical background of gatekeepers).

The present study aims to evaluate the effectiveness of the SUPRA gatekeeper trainings. We expected that the SUPRA gatekeeper trainings would lead to immediate and sustained improvement in knowledge, self-efficacy, attitudes, and behavior relevant for the role as gatekeeper, comparable to existing gatekeeper trainings. Furthermore, we explored if professional experience was associated with effects of gatekeeper trainings, because there was mixed evidence in previous studies (10). Finally, the COVID crisis started in the follow-up period of our study and we explored if this impacted our results.

## 2. Materials and methods

### 2.1. Gatekeeper trainings

#### 2.1.1. Contents, goals, and methods of the SUPRA gatekeeper trainings

The SUPRA gatekeeper trainings are not manualized in a strict sense, but for a 1 day training, the following contents and goals were mandatory: reflection of professional and private experiences with suicidality, training of important gatekeeper skills (e.g., connecting, awareness/handling of transference and counter-transference issues, assessment of suicidality), and providing knowledge. Required topics of knowledge included basic facts of suicide (epidemiology, myths), warning signs, risk factors, theoretical models of suicide, connecting with suicidal people, definition and assessment of acute suicide risk, resources for referral, and crisis intervention. Optional contents, depending on the needs of the audience, included survivors of suicide, suicidality in different life phases, legal aspects, etc. Levels of complexity was adjusted according to the background experience of participants. For example, if the gatekeeper training was held for mental health professionals such as psychiatric nurses or psychologists, more focus was on the psychodynamics of difficult situations, narrative interviewing etc. Different didactic methods were used, such as role-play, case-discussion, group-reflection/discussion, and presentation of slides. The slides were provided by SUPRA, thus allowing standardization of the knowledge-part of the gatekeeper trainings. The mandatory and optional elements of gatekeeper trainings and the didactic principles are described in a manual (13).

The gatekeeper trainings evaluated in this study were planned to last about 8 h each and were part of a curriculum for new trainees to acquire the status as certified gatekeeper trainer. The gatekeeper trainings were led by gatekeeper trainers together with a trainee. Gatekeeper trainers and trainees had a professional background (e.g., medicine, psychology, psychotherapy, social work), at least 5 years of experience in counselling/treatment of suicidal people, and experience with group education. Gatekeeper trainees already participated in a 2 days train-the-trainer curriculum provided by SUPRA senior gatekeeper trainers.

#### 2.1.2. Participants of gatekeeper trainings

There were 28 gatekeeper trainings provided all over Austria between April and November 2019, with 427 participants overall, and a mean number 15.11 participants per training (range 10–21). Gatekeeper trainers or their institutions, who usually had a long-standing tradition in delivering such gatekeeper trainings, did their usual or intensified recruitment of participants in their regions.

### 2.2. Evaluation

#### 2.2.1. Design

The effects of the gatekeeper trainings were assessed with a pre-post-follow-up design, and by comparing the results with those of previous studies serving as benchmarks. Both the pre- and post-assessments were done at the site of the gatekeeper training, immediately before and after the training, with paper- and pencil questionnaires handed out by the trainers. The electronic follow-up assessment was planned 6 months after the day of the gatekeeper training.

#### 2.2.2. Sample size calculation

Sample size calculation was based on the smallest pre-post difference reported in the review by Hangartner et al. (8), and assuming *α* = 0.05 and 95% or 80% power. Consequently, a one-sided *t*-test would require 553 participants for 95% power, or 317 participants for 80% power. According to the plan of the gatekeeper project, we expected about 500 participants. Assuming a pre-post dropout of 10%, and a further post-follow-up dropout of 30%, we expected at least 315 participants remaining for the follow-up assessment, so that the 80% statistical power seemed guaranteed.

#### 2.2.3. Instruments

We opted for the established questionnaire by Wyman et al. (14), because it was used in many previous studies, allowing comparisons and benchmarking, and includes relevant constructs from the theory of planned behavior (11) which are known to correlate with actual intention and behavior (15). The questionnaire consists of scales to assess (declarative) gatekeeper knowledge (e.g., warning signs for suicide), appraisals of self-efficacy (i.e., perceived preparedness for gatekeeper-role, self-evaluation of suicide prevention knowledge, and efficacy to perform gatekeeper-role) and attitudes (i.e., reluctance to engage with suicidal people) as well as gatekeeper behavior in the previous 6 months (e.g., asking depressed people about suicide). According to the theory of planned behavior, the scales assessing perceived preparedness for gatekeeper-role, self-evaluation of suicide prevention knowledge, and efficacy to perform gatekeeper-role correspond to perceived behavioral control, while the scale reluctance to engage with suicidal people assessed attitudes (8).

Items from the instrument by Wyman et al. (14) were translated and back-checked with a native speaking psychologist after obtaining allowance for translation and usage by the original authors. Because the original instrument was developed for schools, we rephrased items to enable usage in all contexts. For the knowledge scale we did not use the original items but developed a set of 10 items that covered the required content of the gatekeeper trainings, which were in line with the information material, and were considered important (see Table 1).

**Table 1:** Scales and items of the assessment instrument, reliability, and use in assessments.

Scales (range of possible values)	Number of items	Reliability (*α*)		Assessment^b^
		Pre^a^	Wyman^c^	
Knowledge items created by authors (0–100%)	10	0.27	—	Pre/post/FU
Epidemiology—suic. Risk and sociodemographics (0/1)	1			Pre/post/FU
Epidemiology—suic. Risk and psychiatric disorders (0/1)	1			Pre/post/FU
Warning signs (0/1)	1			Pre/post/FU
Connecting with suicidal people (helpful aspects) (0/1)	1			Pre/post/FU
Assessing suicidality (helpful questions) (0/1)	1			Pre/post/FU
Assessing suicidality (helpful questions) (0/1)	1			Pre/post/FU
Risk assessment—acute suicidal crisis (0/1)	1			Pre/post/FU
Crisis intervention—contracting (0/1)	1			Pre/post/FU
Crisis intervention—anti-suicide contract (0/1)	1			Pre/post/FU
Crisis intervention—involving relatives/friends (0/1)	1			Pre/post/FU
Knowledge of institutional resources for suic. People (0–1)	4	0.67	0.74	Pre/FU
*Self-efficacy*				
Perceived preparedness for gatekeeper-role (1–7)	8	0.93	0.94	Pre/post/FU
Self-evaluation of suicide prevention knowledge (1–7)	9	0.96	0.97	Pre/post/FU
Efficacy to perform gatekeeper-role (1–7)	7	0.78	0.80	Pre/post/FU
*Attitude*				
Reluctance to engage with suicidal people (1–7)	9	0.57	0.68	Pre/post/FU
*Behavior*				
Asking depressed people about suicide (1–5)	2	0.80	0.77	Pre/FU
Appropriate referral of suicidal people (yes/no)	2	0.76	0.88	Pre/FU
Asking about suicide in response to warning signs (1–5)	4	0.88	0.94	Pre/FU
Use of gatekeeper behaviors with suicidal people (1–5)	7	0.87	0.94	Pre/FU
Personal referral of suicidal people to institutions (0–5)	1		—	Pre/FU

a Reliability based on the pre-assessment.

b Assessment directly before the gatekeeper training (pre), directly afterwards (post), and follow-up (FU) assessment 6 months after the gatekeeper training.

c Reliability as reported in the study by Wyman et al. (14).

For the pre-assessment, a full version of the questionnaire was used, including scales for knowledge, self-efficacy, attitudes, and behavior, as well as sociodemographic variables, professional background, and professional experience (see online Supplementary material). The post-assessment only included scales for knowledge, self-efficacy and attitudes but not behavior, since behavior was assessed for the past 6 months and thus cannot change within 1 day. The follow-up assessment included scales for knowledge, self-efficacy, attitudes, and behavior. Participants generated a personal code allowing linking the three assessments anonymously.

We followed the coding procedure of Wyman et al. (14) to create summary scores of the scales. Because the coding instruction defined no limits for missing data, we created cut-offs of missing data per variable (see online Supplementary material).

The reliability of the scales, as measured with Cronbach’s *α*, were comparable with the original instrument (Table 1). However, the reliability with the knowledge-scale was very low, suggesting that there was no latent knowledge variable. Because the items were considered as important by experts, we analyzed the knowledge-items individually, too.

#### 2.2.4. Statistical analysis

To quantify the effects of the gatekeeper trainings, we calculated differences between the post/follow-up assessments and the pre-assessment, using Cohen’s *d* as a measure of effect size, and one-sided *t*-tests for significance testing, since we expected changes in a specific direction, that is, an improvement in knowledge, self-efficacy, attitudes, and behavior. For binary variables, we used the McNemar test for statistical significance. We also ran mixed-effects regression models but only reported these in the online Supplementary material because results are comparable.

For the short-term effects, we compared our pre-post effects sizes with the benchmarks reported in the review by Hangartner et al. (8) who reported benchmarks on knowledge, self-efficacy (perceived behavioral control), and attitudes.

For the longer-term effects, no related review reporting effect-sizes exists, to our knowledge. Therefore, we selected studies of a recent systematic review of long-term effects of gatekeeper trainings (9) with comparable outcomes and follow-up time-frames of 3 to 6 months. We either used the effect sizes reported in the studies or calculated them based on the means and standard deviations reported in the studies. We calculated the means and bootstrapped 99% confidence intervals of the effect sizes, similar to Hangartner et al. (8), but we used weighted means to account for different sample sizes.

We used R Version 4.0.2 for all data analysis (16). The data, tabulated results from the benchmark studies, the R-code, and additional analyses are available via the Open Science Framework.^1^

#### 2.2.5. Ethical aspects

Participation in the assessments was voluntary. No identifying information was assessed. The email addresses were collected separately. The ethics committee of Salzburg approved the study (Nr. 415-EP/73/807-2019).

## 3. Results

### 3.1. Participants

There were 427 participants according to the sign-up lists and nearly all completed the pre-assessment (*n* = 424, 99%) and the post-assessment (414, 97%). Ninety-three percent of participants (*n* = 398) provided an email address, and the link to the electronic follow-up assessment could be successfully sent to 385 (90%) participants. The follow-up assessment was completed, at least partly, by 135 participants, corresponding to 32% of the 427 signed-up participants and 35% of participants contacted via email. An unambiguous linking with the pre-assessment was possible for 381 (90%) participants of the post-assessment, and for 91 (21%) participants of the follow-up assessment (percentages in brackets based on those who completed the pre-assessment).

Based on the pre-assessment, participants age was *M* = 40.37 (SD = 11.53). Eighty percent (*n* = 339) identified as female, 82 (19%) as male, and 3 (1%) as “something else.” With respect to professional background, 18% were nurses, 16% clinical psychologists, 12% social workers, and 10% counselors. Professional groups with each less than 10% frequency included psychotherapists, supervisors, teachers, administrators, social-pedagogues, psychiatrists, and physicians, and there was missing data for 8 (2%) participants. Around one quarter (26%) of participants were from the mental-health professions where some training in suicide prevention could be expected. Years of experience in the profession was *M* = 10.59 (SD = 9.61, range 0–51).

### 3.2. Changes in knowledge, self-efficacy, attitudes, and behavior

#### 3.2.1. Knowledge

Pre-post comparisons indicated a statistically significant increase of correctly answered knowledge-items (*p* < 0.01), with a large effect size (*d* = 0.83, 95%-confidence interval 0.70–0.96), somewhat smaller compared to previous evaluations of gatekeeper trainings (*d* = 1.22, 0.90–1.52) (Table 2). Pre-follow-up comparisons were statistically significant (*p* < 0.01), with a medium effect size (*d* = 0.60, 0.36–0.83), comparable to previous studies (*d* = 0.62, 0.16–0.95). Analyses of individual items showed statistically significant increases of correct answers for most items in the pre-post comparisons, and for some items in the pre-follow-up comparisons (Table 2). There was a likely ceiling effect for one item about connecting with suicidal people (93%, 94%, and 95% correct answers in the pre-, post, and follow-up assessments, respectively), and there was only minor improvement for two items about warning signs (76%, 70%, 80%) and crisis intervention (27%, 33%, 28%).

**Table 2:** Change of knowledge, attitudes and behavior.

Scale (range of possible values)	Pre	Post	Follow-up	*p*		Effect size pre-post		Effect size pre-follow-up	
	*M* (SD)	*M* (SD)	*M* (SD)	Pre-post	Pre-FU	Cohen’s *d* (95% CI)	Benchmark^b^ (99% CI)	Cohen’s *d* (95% CI)	Benchmark^c^ (99% CI), *n*
Knowledge (0–100%)	57.00 (16.22)	70.05 (15.15)	69.03 (14.19)	0.00	0.00	0.83 (0.70–0.96)	1.22 (0.90–1.52)	0.60 (0.36–0.83)	0.62 (0.16–0.95), 14
Epidemiology—sociodemographics	40	89	84	0.00^a^	0.00^a^				
Epidemiology—psychiatric disorders	42	62	65	0.00^a^	0.00^a^				
Warning signs	76	70	80	0.02^a^	1.00^a^				
Connecting with suicidal people (helpful aspects)	93	94	95	0.50^a^	0.68^a^				
Assessing suicidality (helpful questions)	57	90	93	0.00^a^	0.00^a^				
Assessing suicidality (helpful questions)	85	91	94	0.00^a^	1.00^a^				
Risk assessment—acute suicidal crisis	60	71	68	0.00^a^	1.00^a^				
Crisis intervention—contracting	43	41	28	0.59^a^	0.66^a^				
Crisis intervention—anti-suicide contract	27	33	28	0.02^a^	0.54^a^				
Crisis interv—involving relatives/friends	48	57	59	0.00^a^	0.15^a^				
Knowledge of institutional resources for suicidal people (0–1)	0.63 (0.34)	—	0.79 (0.29)	—	0.00	—	—	0.63 (0.38–0.87)	
*Self-efficacy (1–7)*									
Perceived preparedness for gatekeeper-role	4.51 (1.33)	5.60 (1.11)	5.57 (0.91)	0.00	0.00	0.89 (0.79–0.99)	0.73 (0.19–1.26)	0.93 (0.72–1.13)	0.68 (0.51–1.01), 9
Self-evaluation of suic. prev. knowledge	4.17 (1.39)	5.55 (0.94)	5.59 (0.94)	0.00	0.00	1.09 (0.98–1.20)	0.73 (0.19–1.26)	1.06 (0.84–1.29)	1.12 (0.77–1.33), 9
Efficacy to perform gatekeeper-role	4.45 (1.14)	5.35 (0.97)	5.29 (0.94)	0.00	0.00	0.83 (0.74–0.91)	0.73 (0.19–1.26)	0.72 (0.53–0.92)	0.90 (0.61–1.09), 18
Attitude—reluctance to engage with suicidal people	2.21 (0.64)	1.90 (0.63)	1.95 (0.65)	0.00	0.00	−0.51 (−0.61 to −0.41)	0.06 (−0.54 to 0.65)	−0.29 (−0.53 to −0.06)	−0.37 (−), 1
Behavior		—		—		—	—		
Asking depressed people about suicide (1–5)	3.41 (1.39)	—	3.82 (1.18)	—	0.11	—	—	0.13 (−0.07 to 0.32)	0.13 (−0.04 to 0.20), 7
Asking about suicide in response to warning signs (1–5)	2.79 (1.37)	—	3.25 (1.32)	—	0.03	—	—	0.16 (−0.00 to 0.33)	0.13 (−0.04 to 0.20), 7
Appropriate referral of suicidal people (% yes)	56%	—	68%	—	0.50^a^	—	—	—	—
Use of gatekeeper-behaviors with suicidal people (1–5)	2.78 (1.22)	—	3.26 (1.17)	—	0.00	—	—	0.24 (0.05–0.42)	0.40 (0.22–0.63), 3
Personal referral of suicidal people to institutions (0–5)	0.94 (1.25)	—	1.35 (1.41)	—	0.15	—	—	0.12 (−0.11 to 0.35)	0.23 (0.13–0.27), 6

Boldface are statistically significant results (*p* < 0.05) with large effect sizes (*d* > 0.8). Assessment directly before the gatekeeper training is abbreviates as “pre”, directly afterwards as “post”, and at follow-up as “FU”.

a McNemar test.

b Benchmark results from Hangartner et al. (8).

c Benchmark results from our own calculations, based on Holmes et al. (9), n is the number of available studies/samples for benchmarking.

With respect to subjectively perceived knowledge of institutional resources (only assessed pre and follow-up), there was a statistically significant increase (*p* < 0.01) with a medium effect size (*d* = 0.63, 0.38–0.87).

#### 3.2.2. Gatekeeper self-efficacy and attitudes

For the appraisal of gatekeeper self-efficacy and attitudes, all pre-post and pre-follow-up comparisons were statistically significant (*p* < 0.01). With respect to self-efficacy, for the scale *perceived preparedness for gatekeeper-role*, there was a large pre-post increase (*d* = 0.89, 0.79–0.99), comparable to previous studies (*d* = 0.73, 0.19–1.26). The pre-follow-up difference was also large (*d* = 0.93, 0.72–1.13), larger than the average effect of other evaluations of gatekeeper trainings (*d* = 0.68, 0.51–1.01), but the confidence intervals overlapped.

The scale assessing *self-evaluation of suicide prevention knowledge* had a large pre-post increase (*d* = 1.09, 0.98–1.20), larger than in previous studies (*d* = 0.73, 0.19–1.26), but confidence intervals overlapped. The pre-follow-up difference was also large (*d* = 1.06, 0.84–1.29) and comparable with findings from previous studies (*d* = 1.12, 0.77–1.33).

Subjectively experienced *efficacy to perform gatekeeper-role* increased pre-post with a large effect size (*d* = 0.83, 0.74–0.91), comparable to previous studies (*d* = 0.73, 0.19–1.26). The pre-follow-up difference was also medium to large (*d* = 0.72, 0.53–0.92), comparable to previous studies (*d* = 0.90, 0.61–1.09).

Finally, with respect to attitudes, *reluctance to engage with suicidal people* decreased with a medium effect size (*d* = −0.51, −0.61 to −0.41), larger than in previous studies but with overlapping confidence intervals (*d* = 0.06, −0.54 to 0.65). The pre-follow-up difference had a small to medium effect size (*d* = −0.29, −0.53 to −0.06). There was only one study for comparison, with a comparable effect (*d* = −0.37).

#### 3.2.3. Behavior

*Asking depressed people about suicide* did not significantly increase from the pre- to the follow-up assessment (*p* = 0.11). The effect size was small (*d* = 0.13, −0.07 to 0.32). In contrast, *asking about suicide in response to warning signs* increased statistically significant pre-follow-up (*p* = 0.03). The effect size was small (*d* = 0.16, −0.00 to 0.33). Both results were comparable with those from previous studies (*d* = 0.13, −0.04 to 0.20).

Appropriate *referral of suicidal people* increased from 56 to 68%, but the difference was not statistically significant (*p* = 0.50). Number of *suicidal people referred personally to institutions* increased slightly but not statistically significant (*p* = 0.15), with a small effect size (*d* = 0.12, −0.11 to 0.35). Previous studies reported somewhat larger effects (*d* = 0.23, 0.13–0.27), but the confidence intervals overlapped.

The *use of gatekeeper-behaviors with suicidal people* increased statistically significant (*p* < 0.01), with a small effect size (*d* = 0.24, 0.05–0.42), somewhat smaller than in previous studies (*d* = 0.40, 0.22–0.63), but with overlapping confidence intervals.

### 3.3. Additional and sensitivity analyses

#### 3.3.1. Associations of differences with professional experience

Years of professional experience were not or only weakly associated with pre-post changes of knowledge, self-efficacy, or attitudes (all *r* < 0.15). Similarly, pre-follow-up differences in knowledge, self-efficacy, attitudes or behavior only weakly correlated with professional experience (all *r* < 0.22), with two statistically significant exceptions: more years of professional experience was positively associated with increase of the number of suicidal people personally referred to institutions (*r* = 0.28, *p* = 0.01) and less increase of felt preparedness (*r* = −0.23, *p* = 0.04).

#### 3.3.2. Representativeness of the follow-up assessment

With respect to improvements in knowledge, self-efficacy, attitudes, and behavior, there were only minor differences between the 91 participants whose follow-up assessment could be linked unambiguously to their pre-assessment and the other participants (all *d* < 0.19). However, there was one exception: participants without a follow-up assessment had significantly larger pre-post reductions in negative attitudes, compared to participants with a follow-up assessment (*p* = 0.01, *d* = 0.29). With respect to age, gender, and professional experience, there were no significant differences between those with and without a follow-up assessment, and all differences were small (*d* < 0.21). The same applied to knowledge, attitudes, and behavior at the pre- or post-assessments. Further details can be found in the online Supplementary material.

#### 3.3.3. Effects of the COVID-19 pandemic on the follow-up assessment

The COVID-19 pandemic started between the first and last follow-up assessments, with a national lockdown on March 16, 2020, to May 1, 2020. Many treatment facilities had restricted availability or were closed and there was a substantial drop in psychiatric admissions. This could have impacted our results. However, after the national lockdown, the response was only slightly lower than before (20 vs. 26%), and the difference was not statistically significant (OR = 0.70, 95% CI 0.43–1.14, *p* = 0.15). The improvement of gatekeeper-behavior was numerically larger before the lockdown than compared to afterwards, with small to medium effect sizes but the differences were not statistically significant.

## 4. Discussion

In this study, we evaluated the effects of the SUPRA gatekeeper trainings on knowledge, self-efficacy, attitudes, and behavior in the short- and longer-term. For the short-term improvements assessed immediately after the gatekeeper trainings, we observed medium to large improvements of knowledge, but somewhat smaller than in previous studies. There were large improvements of self-efficacy and attitudes (preparedness for gatekeeper role, self-evaluation of knowledge, efficacy to perform gatekeeper-role, and reluctance to engage with suicidal people), comparable or larger than in previous studies. For the longer-term improvements assessed 6 months after the gatekeeper training, most of the short-term effects for knowledge, self-efficacy and attitudes upheld and were comparable or larger than in studies of established gatekeeper programs. Increase of gatekeeper behavior was generally small (*d* < 0.30) but in line with evaluations of other gatekeeper trainings.

The substantial and enduring effects for knowledge, self-efficacy and attitudes may be explained by the interactive nature, flexible and multi-method approach, and length of the gatekeeper training, which perhaps allowed learning and attitude change in more depth. In line with this assumption, Condron et al. (17) found that a 2 days gatekeeper training with experience-based learning possibilities lead to more changes, compared to a gatekeeper training of only a few hours, at least for certain participants and outcomes. SUPRA gatekeeper training were not manualized in a strict sense but mandatory elements had to be included. We did not assess how the gatekeeper trainings actually varied in didactic methods and adherence to content and how this affected outcome or satisfaction with training by participants and trainers. However, similar limitations may apply to already existing gatekeeper trainings which are more strictly manualized. From what is known in psychotherapy research, manualization is not associated with superior outcome (18) but it is not sure how this applies to gatekeeper trainings. More research about adherence and outcome in gatekeeper trainings is needed, as well as dismantling studies for content and didactic methods.

In contrast to knowledge/self-efficacy/attitudes, the improvements of gatekeeper behavior were small, but the results are in line with previous studies according to our benchmark analysis (8).

The results in our study are in line with the assumptions of the theory of planned behavior, where self-efficacy, attitudes, and subjective norms (which we did not assess in our study) determine actual behavior. However, an empirical test of this theory was beyond the scope of this paper and also not possible because we had no experimental design with a control group.

The effects of the training were largely independent of the wide range of different professional backgrounds and experience of participants in the gatekeeper trainings. This is noteworthy, because about one quarter of participants were mental health professionals, where it can be expected that there is already is suicide preventive expertise and also that there is thus less improvement after gatekeeper trainings. However, other studies found mixed evidence for limited effects of gatekeeper trainings provided to mental health professionals compared to other health professionals (10). We found only one small significant association between years of professional experience and increase of the number of suicidal people personally referred to institutions. This is counterintuitive and may also be a false positive finding due to multiple testing.

Finally, the COVID pandemic did not seem to have notably distorted the study results, despite that the follow-up assessment period overlapped with the national lock-down in response to the COVID pandemic. This might have biased the results because many institutions closed or restricted their capacity, potentially leading to fewer opportunities for gatekeeper behavior and lower response rate of the follow-up assessment. However, sensitivity analysis revealed no substantial difference in gatekeeper behavior or response rates before and after the lock-down, perhaps due to compensatory outreach to clients and patients electronically or by phone.

### 4.1. Limitations and strenghts

A main limitation of our study is that there was no control group. Instead, the effects of the gatekeeper program were compared with the benchmark effects of other gatekeeper trainings. Benchmarking is an alternative approach that can provide valuable information on effectiveness (19). Our Benchmarks were based on two existing systematic reviews (8, 9) and thus avoided cherry picking of research. However, our approach has limitations, mainly because it was based on studies which are heterogeneous with respect to trainings, target populations, length of training, or measures, and these differences may be associated with the outcome. Furthermore, the number of studies was too small for subgroup-analyses and in our trainings there was a variety of participants and settings which we did not systematically assess, thus preventing a more fine-grained comparison. The question of how variables such as training method, assessment, setting, and participants impacts the outcome should be the topic of future systematic reviews. Moreover, the systematic reviews we used for comparing our results with those of existing studies may have missed some relevant research (20, 21) because of the search strategy or because new research appeared since then. For the comparison of attitudes, the short-term benchmarks are not specified in detail by Hangartner et al. (8), and there was only one study for the long-term benchmarking. One more potential limitation is that the SUPRA gatekeeper trainings were led by very experienced trainers together with trainees. Thus, it still has to be demonstrated that the effects observed in our study translate to future gatekeeper-trainings held by less experienced gatekeeper trainers.

Another limitation in our and in most existing related studies is that assessments were based on self-reports only and that self-reported improvements of attitudes and behavior may not correlate well with actual improvements of gatekeeper behavior. However, according to the theory of planned behavior and the large and enduring changes in knowledge, self-efficacy, and moderate changes in attitudes in our study, it is plausible that the gatekeeper trainings lead to at least some actual changes in behavior. Investigating objectively observable change induced by gatekeeper trainings needs studies that are much more difficult to do, for example randomized controlled trials with observations of actual gatekeeper behavior with actors playing suicidal people or by investigating actual referral rates in administrative data. It is thus not surprising that there are only few such studies (22–24). Furthermore, as one reviewer pointed out, our instrument (14) was specifically developed for the question-persuade, refer (QPR) curriculum (25) and perhaps was not optimal for the SUPRA gatekeeper trainings.

Even if gatekeeper trainings substantially improve gatekeeper behavior, it is still an open question if this leads to a notable reduction of suicide rates (26). Again, this needs more complex research designs, for example, cluster-randomized designs, where regions are supported with gatekeeper trainings or not, and observing suicide rates in these regions before and after the gatekeeper trainings. One study found significantly reduced suicide rates in regions with gatekeeper trainings, relative to control regions (27). In contrast, a randomized controlled study could not find reduced suicide attempt rates associated with the implementation of gatekeeper trainings for teachers and other school personnel (28). However, a suicide prevention program with peers as gatekeeper was associated with reduced suicide attempts in several studies (29).

Another potential limitation is that we translated the assessment instrument into German for the first time without knowing if we achieve satisfactory psychometric properties. However, the reliability of the scales for attitude and behavior was comparable to the original instrument (14) and ranged from satisfactory to excellent. Validating our translated instrument with other instruments and populations may be a next step. Another limitation was that the reliability of the knowledge scale was not satisfactory. Similarly, previous studies also reported rather low reliability for instruments assessing gatekeeper knowledge (30, 31). In our study, we selected knowledge items deemed as important by the expert group, but these items did not correlate high with each other, and some items had a ceiling effect. The low reliability may indicate that we assessed very different aspects of knowledge. Similarly, the reliability of the scale about reluctance to engage with suicidal people was low, calling for improvement.

Finally, whereas it was a strength that rates of response and successful linkage of the assessments for each participants was high in the pre- and post-assessments, the study was potentially limited by a much lower response than expected for the follow-up assessment. Fortunately, most observed effects were large and lacking statistical power was thus not a major issue. Furthermore, there were only marginal differences between participants with and without completion of the follow-up assessment. The only significant finding was that participants without a follow-up assessment had significantly larger pre-post reductions in attitudes, compared to participants with a follow-up assessment, thus the long-term results might have been better with less drop-out.

## 5. Conclusion

Gatekeeper trainings of the national suicide prevention program in Austria had positive and enduring effects on knowledge, self-efficacy, and attitudes, comparable or larger than found in previous studies of other gatekeeper programs. Increases in self-reported gatekeeper behavior were small, but this is in line with results from other gatekeeper programs. Future research is needed to clarify if gatekeeper trainings actually change gatekeeper behavior and reduce suicidal behavior in the population.

## Data availability statement

The original contributions presented in the study are publicly available. This data can be found here: https://osf.io/9bazp/.

## Ethics statement

The studies involving human participants were reviewed and approved by the ethics committee of Salzburg (Nr. 415-EP/73/807-2019). The patients/participants provided their written informed consent to participate in this study.

## Author contributions

MP, TK, and US contributed to conception, design of the study, and data-acquisition. CF and MP reviewed the literature, wrote the manuscript, and performed the statistical analysis. CF, MP, TN, TK, and US drafted and reviewed all the draft versions of the manuscript. All authors contributed to the article and approved the submitted version.

## Funding

This research was funded by PHARMIG Austria.

## Conflict of interest

The authors declare that the research was conducted in the absence of any commercial or financial relationships that could be construed as a potential conflict of interest.

## Publisher’s note

All claims expressed in this article are solely those of the authors and do not necessarily represent those of their affiliated organizations, or those of the publisher, the editors and the reviewers. Any product that may be evaluated in this article, or claim that may be made by its manufacturer, is not guaranteed or endorsed by the publisher.

## Footnotes

^1^ https://osf.io/9bazp
