# Supplementary material for: Effects of gatekeeper trainings from the Austrian national suicide prevention program
Source: Front Psychiatry. 2023 Jul 20;14:1118319. doi: 10.3389/fpsyt.2023.1118319 (PMC10397513; doi:10.3389/fpsyt.2023.1118319)
Supplement: Supplementary file 1 [file Table_1.pdf]

Supplementary Table 1

Missing Data

Subscales	n (%)		
	pre	post	FU
Knowledge items created by authors	0 (0)	44 (10)	345 (81)
Knowledge of institutional resources for suic. people	0 (0)	-	346 (82)
Self-efficacy			
Perceived preparedness for GK-role	6 (1)	43 (10)	340 (80)
Self-evaluation of suicide prevention knowledge	6 (1)	60 (14)	342 (81)
Efficacy to perform GK-Role	4 (1)	55 (13)	345 (81)
Attitude - Reluctance to engage with suicidal people	0 (0)	52 (12)	345 (81)
Behavior			
Asking depressed people about suicide	61 (14)	-	353 (83)
Appropriate referral of suicidal people	112 (26)	-	362 (85)
Asking about suicide in response to warning signs	0 (0)	-	346 (82)
Use of GK-behaviors with suicidal people	0 (0)	-	347 (82)
Personal referral of suicidal people to institutions	2 (0)	-	346 (82)

Note:

Missing data in the pre-assessment is solely due to missed responses. Missing data in the post- and FU-assessments are both from missed responses, not taking part in the assessment (e.g., due to technical problems such as wrong email or refusing to take part).

Supplementary Table 2

Cut-off for missing data

Subscales	Number of Items	Cut-Off
Knowledge items created by authors	10	7
Attitudes		
Perceived preparedness for GK-role	8	6
Self-evaluation of suicide prevention knowledge	9	7
Efficacy to perform GK-Role	7	6
Reluctance to engage with suicidal people	9	7
Behavior		
Asking depressed people about suicide	2	2
Appropriate referral of suicidal people	2	2
Asking about suicide in response to warning signs	4	3
Use of GK-behaviors with suicidal people	7	5

Note:

Cut-offs are the minimum number of missing data which resulted in a missing value for the subscale

Additional analysis

Additional analyses mentioned in the paper are provided in the R-Output which is located here (file "Results.html"): https://osf.io/9bazp/
